# Cutaneous Manifestations Associated with Diabetes Mellitus—A Retrospective Study

**DOI:** 10.3390/diseases11030106

**Published:** 2023-08-18

**Authors:** Dan Vâță, Diana-Elena Stanciu, Doinița Temelie-Olinici, Elena Porumb-Andrese, Bogdan-Marian Tarcău, Vasile-Bogdan Grecu, Laura Gheucă-Solovăstru

**Affiliations:** 1 Department of Dermatology, “Grigore T. Popa” University of Medicine and Pharmacy, 700115 Iași, Romania; danvata@yahoo.com (D.V.); andrese.elena@yahoo.com (E.P.-A.); bogdan.tarcau@yahoo.com (B.-M.T.); lsolovastru13@yahoo.com (L.G.-S.); 2 Dermatology Clinic, “St. Spiridon” Emergency County Hospital, 700106 Iasi, Romania; 3 Department of Cell Biology, “Grigore T. Popa” University of Medicine and Pharmacy, 700115 Iași, Romania; grecu_bogdan@ymail.com

**Keywords:** diabetes mellitus, cutaneous manifestations, acanthosis nigricans, necrobiosis lipodica

## Abstract

Diabetes mellitus (DM) is one of the world’s most important health problems, affecting more than half a billion of the world’s population today, with an ever-increasing prevalence. Among the most common manifestations of diabetes are skin manifestations, with 30–70% of patients experiencing skin complications during the course of the disease. Conditions such as acanthosis nigricans, diabetic dermopathy, necrobiosis lipoidica, bacterial infections, fungal infections, skin xerosis, and metabolic prurigo are often associated with diabetes and often precede its diagnosis. We conducted a retrospective study on a group of 103 patients hospitalized between January 2018 and December 2022, in a clinic of a county hospital, using as criteria the diagnosis of diabetes mellitus complicated by cutaneous manifestations frequently associated with diabetes. The aim was to observe which are the most common manifestations and whether they correlate with data in the research literature. In the present study, manifestations such as diabetic foot (20% of patients), bacterial (35%) and fungal infections, and cutaneous xerosis (45%) were predominant. Often, the integumentary involvement may precede the diagnosis of the underlying disease. It is therefore very important to recognize, investigate and treat these manifestations as soon as possible.

## 1. Introduction

Diabetes mellitus is one of the major global health problems, both because of its increasing prevalence and the complexity of its systemic and local manifestations. Diabetes affects more than half a billion of the world’s population today and is present in more than 10.5% of the adult population. The manifestations of diabetes are very varied, among the most numerous being those at the cutaneous level, so the dermatologist has a very important role in detecting these associations. Conditions such as acanthosis nigricans, diabetic dermopathy, lipoid necrobiosis, bacterial and fungal infections, and skin xerosis are frequently associated with diabetes, often preceding its diagnosis [1,2].

The World Health Organization describes diabetes mellitus as a metabolic disorder of diverse etiology, characterized by chronic hyperglycemia with disturbances in protein, carbohydrate and fat metabolism, resulting from impaired insulin action, insulin secretion or both. Between 30 and 70% of patients with DM (both type 1 and type 2) will develop a skin complication at some point in the course of the disease [1,2,3,4]. The skin manifestations of diabetes have a varied impact on health, ranging from cosmetic changes to life-threatening manifestations [1,3].

According to the most recent data [2], in 2021 the prevalence of diabetes worldwide for the population aged 20–79 years was estimated at approximately 536.6 million people (10.5%), and could reach more than 780 million people (12.2%) in 2045. A higher prevalence has been observed in urban areas (12.1%) and high-income countries compared to rural areas (8.3%) and low-income countries [2,4,5].

The relationship between DM and skin disorders can be divided into: cutaneous manifestations strongly associated with DM, non-specific dermatological signs and symptoms associated with DM, dermatological disorders associated with DM, frequent skin infections occurring in DM and skin changes associated with DM therapy (Table 1) [1,5,6].

Various skin infections are frequently observed in patients with DM: bacterial-erysipelas, cellulitis, erythrasma, necrotizing fasciitis, or fungal-cutaneous or mucous membrane candidiasis, dermatophytosis (tinea pedis) [4]. Medication given in the treatment of diabetes can also have an effect on the skin. Changes that may occur following insulin administration are lipohypertrophy/lipoatrophy at the injection site, allergic reactions, pruritus, induration of the integument, erythema, calcifications. As for oral medication, oral antidiabetics can have numerous cutaneous adverse reactions such as polymorphic erythema, bullous dermatoses (bullous pemphigoid), Stevens–Johnson syndrome, angioedema, allergic reactions, photosensitivity [4,5,6].

### 1.1. Manifestations Strongly Associated with DM

Acanthosis nigricans is a common manifestation in diabetes mellitus, more common in type 2, with a clinical appearance of imprecisely demarcated, dark brown or grey plaques/placards and a thickened, warty surface, predominantly arranged in folds, usually asymptomatic. Prevalence by gender is approximately equal, but occurs more often in patients with dark phototype [1]. It has been observed in several endocrinopathies associated with insulin resistance (acromegaly, Cushing’s syndrome, obesity, thyroid dysfunction, polycystic ovary syndrome), but can also occur in malignancies, not correlated with diabetes, frequently in gastric cancer [7].

In terms of pathogenesis, this is incompletely elucidated, an activation of insulin-like growth factor-1 (IGF-1) receptors in keratinocytes and fibroblasts being incriminated, which leads to cell proliferation, these changes being induced by hyperinsulinemia [8,9].

Treatment involves the adoption of general measures-weight loss, physical activity, proper diet. Glycemic control through the introduction of oral antidiabetics in patients with DM helps to improve the clinical appearance of AN. In case of fissures or hyperkeratotic areas, oral retinoids or topical keratolytic agents (salicylic acid, retinoic acid) can be used [5].

**Figure 1:**
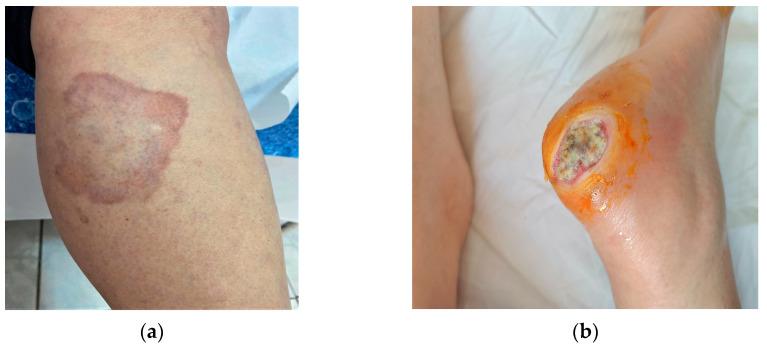
Manifestations strongly associated with DM: (**a**) necrobiosis lipoidica; (**b**) diabetic foot-chronic ulcers.

Diabetic foot (Figure 1b) includes vascular and neuropathic complications that develop in patients with DM, the manifestation being slightly more prevalent in patients with type 1 DM. Clinically, callosities, xerosis, evolving to chronic ulcers and foot malformations occur in the initial stage. Ulcerations (perforating foot ulceration) develop on pressure areas subject to frequent trauma. They heal poorly, are prone to bacterial or fungal superinfection, and if they do heal, recurrence is common. The factors leading to these changes are a combination of neuropathy, atherosclerosis and poor healing. Treatment includes rigorous hygiene, wearing appropriate footwear to minimize and redistribute plantar pressure. In case of superinfection, antibiotics should be administered and special hydrogel dressings, topical growth factors or grafts for epithelization. In ischemic ulcers, surgical revascularization should be used [10,11].

Necrobiosis lipoidica (NL) (Figure 1a) is a rare granulomatous dermatological condition, predominantly in females. NL may be associated with both types of diabetes mellitus. It occurs predominantly in patients with type 1DM in the 3rd decade of life. Risk factors associated with NL are hypertension, hyperlipidemia, obesity, smoking, thyroid dysfunction, kidney disease. The clinical appearance is round erythematous papules, converging into plaques with purplish-red margins and an atrophic, sclerosing, yellow-brown center with telangiectasias on the surface. They occur on the calves, bilaterally in the pretibial region, less commonly on the forearms, scalp, face or abdomen. They are often asymptomatic, but may sometimes present with itching and hypoesthesia or pain if ulceration occurs. Ulcerations occur in 1/3 of cases and are complicated by secondary bacterial infections or progress to squamous cell carcinomas. Some hypotheses on the etiopathogenesis are represented by diabetes-associated microangiopathy, alterations in collagen, neutrophil chemotaxis or blood vessels. Treatment is complex with variable success rates. Topical, intraletional or systemic corticosteroids, are administered in the active phases. Other therapeutic options are calcineurin inhibitors, TNF-alpha inhibitors, pentoxifylline, synthetic antimalarials, PUVA. For ulcerated and bacterially superinfected lesions, local antibiotics, emollients, compressive bandaging are used [4,11,12].

Another specific manifestation of DM is diabetic bullae (bullosis diabeticorum), characterized by the spontaneous appearance of tension bullae on the integument that are usually painless, open quickly and leave erosions, but heal without scarring. It progresses to spontaneous healing but requires prevention of bacterial superinfection [13].

### 1.2. Non-Specific Signs and Symptoms Associated with DM

Acrochordons are soft, pedunculated pseudotumoral formations, having the color of the surrounding integument or hyperpigmented, benign, located in the axillary, cervical, crural or palpebral area. When multiple, they are frequently associated with impaired glucose metabolism, probably due to insulin-induced keratinocyte proliferation. A correlation has been observed between the number of acrochordons and blood glucose levels has been observed. Treatment is aesthetic and can be performed by excision, electrotherapy or cryotherapy [14].

Eruptive xanthomas are reddish-yellow papules arranged in a clustered manner on erythematous tegument and are associated with elevated serum triglyceride levels involving accumulation of chylomicrons and VLDL. They are frequently located on the extensor surfaces of the extremities or in the buttock region and are asymptomatic. Therapy consists of controlling lipid metabolism by diet or specific medication. Short-term treatment may consist of excision, curettage or laser therapy [15].

Skin itching is one of the most common symptoms that occur in diabetic patients being frequently caused by xerosis. It is sometimes associated with the presence of erythematous papules or nodular prurigo. Sympathetic nerve dysfunction, with impaired sweat function, are factors contributing to hypohidrosis and skin dryness. Another hypothesis holds that destruction of sensory c-fibers following diabetic polyneuropathy contributes to pruritus. Treatment involves normalizing serum glucose levels and topically applying urea emollients or anti-pruritic substances (menthol, calamine). In more severe cases, which associate nodular prurigo, topical corticosteroids (mometasone furoate/betamethasone dipropionate) may be applied. Systemic antihistamines, as well as some antidepressants (given in the evening) in refractory cases [16] are also necessary.

Rubeosis faciei occurs frequently in patients with DM and is a sign of poor glycemic control. Chronic hyperglycemia leads to impaired microcirculation, which also becomes clinically obvious by dilation of the facial vessels. Rubeosis also involves microangiopathy that may be present at other levels, so the patient should be evaluated systemically. Treatment consists mainly of glycemic control [15].

### 1.3. Other Dermatological Conditions Associated with DM

Granuloma annulare is a dermatological pathology manifested by the appearance of erythematous papules confluent in plaques and placards with more active, purplish-red margins and a paler, non-atrophic center, usually arranged on the dorsal surfaces of the legs, hands or joints. The generalized form is associated with various comorbidities-malignancies, viral hepatitis B or C, thyroid disease, HIV infection or diabetes. Lesions are asymptomatic or pruritic and heal with post-inflammatory hypo- or hyperpigmentation. As treatment, topical or intralesional corticosteroids, calcineurin inhibitors, PUVA therapy can be administered. Agents such as pentoxifylline or synthetic antimalarials (hydroxychloroquine) have been shown to be effective in the treatment of granuloma annulare [16].

Lichen planus, another condition with incompletely elucidated etiology, has been observed to be associated with diabetes mellitus, with 25% of patients with LP also suffering from DM. Clinically it is manifested by the presence of erythemato-violaceous, pruritic, polygonal papules with whitish, shiny streaks (Wickham striae) on the surface, frequently located at the wrist and ankle joints, sometimes pruritic. Treatment consists of topical corticosteroids, calcineurin inhibitors, phototherapy or systemic administration of corticosteroids, methotrexate, dapsone, hydroxychloroquine [17,18].

In addition, other dermatological conditions have been reported in the literature that may be associated with DM, with varying frequencies: vitiligo [1], psoriasis [18], hidradenitis suppurativa [19,20].

### 1.4. Common Skin Infections in Patients with DM

Patients with DM are more susceptible to skin infections due to hyperglycemia-induced metabolic and immunological alterations. It has been observed that in these patients, skin pH is higher, promoting bacterial colonization. Additionally, vascular changes and diabetic neuropathy lead to a lower perception of pain, thus patients do not feel local trauma, which is complicated by superinfection and ultimately slow wound healing. Over 50% of patients with DM will at some point in the course of the disease present with an infectious episode [5]. Common bacterial infections include agents such as *Staphylococcus aureus*, (involved in the development of folliculitis, abscesses, impetigo), group A beta-hemolytic staphylococci (which can cause ecthyma, erysipelas/cellulitis, necrotizing fasciitis) [21].

Fungi are another class of pathogens often involved in skin infections in diabetic patients. Cutaneo-mucosal candidiases are common among patients suffering from DM, especially recurrent infections with Candida albicans, which are difficult to cure and may point us towards a hyperglycemic status of patients [22]. Additionally, this category includes dermatophytoses, which affect the skin and nails, manifesting as tinea pedis (hyperkeratotic or intertriginous) and onychomycosis. Fissures in tinea pedis represent portals of entry for bacterial agents, leading to local complications and slow, poor healing [23].

### 1.5. Cutaneous Complications Following Antidiabetic Therapy

Skin reactions caused by insulin therapy: lipohypertrophy (most common manifestation, at the site of insulin injection), lipoatrophy, erythema, local infections, subcutaneous nodules, allergies [24]. Lipohypertrophy is the most common local reaction after insulin injection, affecting approximately 27% of patients. Adipocyte hypertrophy occurs at the injection site, most likely caused by activation of adipocytes by insulin. This complication may affect local insulin absorption [25].

Lipoatrophy is characterized by an atrophy of subcutaneous adipose tissue at the site of insulin injection, the incriminated cause being the activation of an inflammatory cascade due to vascular deposits of immunoglobulins, leading to a blockage in the physiology of adipocytes [26]. Reactions induced by oral antidiabetics (gliptin) are rare and manifest as drug eruptions, polymorphic erythema, leukocytoclastic vasculitis, photosensitivity, rare—pemphigus vulgaris/bullous pemphigoid. The drugs frequently incriminated belong to the sulphonylurea class (glibenclamide, tolbutamide, chlorpropamide), which, according to some authors, also induce psoriatic or lichenoid eruptions. The occurrence of pemphigus vulgaris has also been reported very rarely [4,24].

Another substance that has induced skin reactions is metformin, a biguanide that can cause leukocytoclastic vasculitis, psoriatic eruptions. Acarbose, an alpha-glucosidase inhibitor, has been incriminated in the development of erythema multiforme and generalized exanthematous pustulosis [4,24].

## 2. Materials and Methods

In order to achieve the objectives of the present retrospective study, the case reports registered in the dermatology clinic between 2018 and 2022 were analyzed. This revealed a total of 427 patients with DM: 6 patients with DM type 1 and 421 patients with DM type 2. Of these, only patients (n = 103) with diabetes who presented at the time of admission with at least one of the cutaneo-mucosal manifestations associated with this condition were considered eligible. Our aim was to determine which were the most prevalent manifestations and to compare the results with the ones already existent in the literature.

The data were centralized in a 24.0 database SPSS and processed with the statistical functions which they are suitable, at the significance threshold of 95%. The Chi-Square Test is a non-parametric test comparing 2 or more frequency distributions from the same population; applied when expected events are excluded.

The data were analyzed and summarized in Table 2, Table 3, Table 4, Table 5, Table 6 and Table 7 with the afferent explanations. We selected the cutaneous and mucosal manifestations in DM from articles on the same subject, analyzing them in our patients.

## 3. Results

The distribution by gender and age groups (Table 1) was of 58% female (60 patients) and 42% male (43 patients). Patient ages ranged from 32 to 88 years, with a mean of 63.3 years. The 60–69 years age group 37 patients (35.9%) prevailed, followed by the ≥70 years age group with 32 patients (31.1%), and on the 3rd place came the 50–59 years age group with a total of 25 patients (24.2%). The 40–49 years age group included 7 patients (6.8%), and on the last place, the 30–39 years age group with 2 patients.

The Chi-Square Test-Likelihood Ratio revealed no significant differences by age and sex distributions (*p* = 0.637).

According to diabetes type, type 2 diabetes predominated (98.1%-101 patients), with only 1.9% having type 1 DM (2 patients), no differences between sexes (*p* = 0.812) (Table 2).

In terms of manifestations strongly associated with DM (Table 3), diabetic foot was predominant in 18.4% of patients, occurring more frequently in males (63%) (*p* = 0.037). It was followed by necrobiosis lipoidica in 6.8% of patients (0.466), of which 71% in females. Bullosis diabeticorum was present in 5.8% of patients (*p* = 0.204), followed by acanthosis nigricans in 4.9% of patients (*p* = 0.077), also predominantly in males (80%).

In the analysis of signs and symptoms commonly associated with DM, the highest percentage was recorded in the case of skin xerosis, found in 44 patients (42.7%), of which 64% females (*p* = 0.341), followed by pruritus, present in 15.5% of patients (*p* = 0.203). Acrochordons were present in 14.5% of patients (*p* = 0.327), and rubeosis faciei was present in equal numbers in both sexes, amounting to 5.8% (*p* = 0.674).

In the case of skin and mucosal infections associated with DM (Table 5), bacterial infections predominated, occurring in 33.9% of patients, with cellulitis, folliculitis and erythrasma being common. With an almost equal number of cases, tinea pedis occurred in 31.1% of patients, with a slight male predominance (53%; *p* = 0.118), followed by cutaneous mycoses (most frequently crural/axillary/submammary intertrigo) in 27.1% of cases, mainly present in females (71%; *p* = 0.099). The lowest percentages were recorded by oral candidiasis (13.6%; *p* = 0.624) and genital candidiasis in 6.8% of patients (*p* = 0.101), whithout differences between sexes.

Table 6 shows the distribution of other dermatological conditions that can be associated with DM. The association of bullous autoimmune conditions such as pemphigoid bullosa, pemphigus vulgaris, dermatitis herpetiformis, morphea was observed in 19 patients (18.4%), whithout differences between sexes (*p* = 0.633), also without differences between sexes, 10.6% of patients with DM also associated psoriasis (*p* = 0.703), followed by 8.7% with lichen planus (*p* = 0.594). Another associated condition was granuloma annulare in 4 patients (predominantly in female), but procentual distribution by sexes are not significance (*p* = 0.491).

## 4. Discussion

In general, the cutaneous manifestations of diabetes appear after the development of the disease, but they may also be the first signs or even precede the manifestations of the primary condition by several years. The prevalence of skin involvement does not appear to differ between patients with type 1 and type 2 diabetes. However, it has been found that patients with type 2 diabetes develop skin lesions associated with infections more frequently, whereas patients with type 1 diabetes often have autoimmune skin lesions [1,4,11].

Cutaneous manifestations in diabetes mellitus are varied in nature, appearance and location, but are not always conditioned, as in other metabolic disorders, by elevated blood glucose levels. Sometimes it is even possible to speak of hyperglycemia (cutaneous diabetes) dissociated from hyperglycemia [1,4,11].

There are several mechanisms by which constant hyperglycemia induces changes in the skin. Elevated plasma glucose levels can affect cells both directly and through advanced glycosylation end-products (AGEs). The activity of keratinocytes and fibroblasts can be directly affected by hyperglycemia through changes in protein synthesis, proliferation and migration. Dysfunction of vasodilation also occurs through inhibition of nitric oxide molecules. Furthermore, mitochondria are damaged as a result of sorbitol overregulation, ultimately leading to the release of reactive oxygen species [2,4,5]. As a result of non-enzymatic reactions of glucose with other molecules (proteins, lipids, nucleotides) AGEs are formed, which bind to specific receptors and ultimately promote the production of pro-inflammatory cytokines. AGEs can also induce the production of free radicals, causing oxidative stress [4,5]. At the same time, certain reactions of AGEs with collagen type 1 or epidermal growth factor (EGF) can lead to suppression of integument regeneration [4,5].

Hyperglycemia leads to non-enzymatic glycosylation of many structural and regulatory proteins, including collagen. Although non-enzymatic glycosylation typically occurs with advancing age, the process is greatly accelerated in diabetes. Non-enzymatic glycosylation leads to the formation of advanced glycosylation end products, which are responsible for decreased acid solubility and enzymatic digestion of collagen in the skin. Alterations, such as the diabetic’s thickened skin and limited joint mobility, are thought to be a direct result of the accumulation of advanced glycosylation end products. Studies show that the level of advanced glycosylation end products in the skin is strongly correlated with retinopathy, nephropathy and other microvascular complications of diabetes [2,5,20,21,22].

The pathological effects of advanced glycosylation end-products consist, firstly, of the fact that the substances thus modified lose some of their properties, and secondly, a number of cells, such as endothelial cells, adipocytes, monocytes, possess surface receptors for advanced glycosylation end-products. By binding advanced glycosylation products to these receptors, oxidative stress and inflammation are stimulated and reactions are triggered, ultimately giving rise to endothelial and adipocyte dysfunction [2,5,20,23].

In the present study, carried out on a group of 103 patients, we could observe a prevalence of these manifestations in age groups over 50 years, with a mean age of 63.3 years and a predominance of female gender-60 female, 43 male (F:M = 1.4:1). Original articles on the same topic have included similar groups in terms of number of patients and mean age, such as the study carried out by Goyal et al. [27] which included 100 patients with a mean age of 57.4 years, in whom the most common manifestations (xerosis—44 patients, infections—31 patients, and rubeosis faciei—4 patients) are comparable in percentage to those found in our study. In terms of gender distribution, however, studies by Garg et al. [3] and Goyal et al. [27] show a predominance of males (55% males, 45% females in the first study and 54% males, 46% females in the second study) compared to the present study where females predominated. Comparable results were found in a study carried out by Timshina et al. [28], where the female to male ratio was 1.21:1.

Regarding the type of diabetes, 98% had type 2 DM, while only 1.94% had type 1 DM, which is consistent with other studies, such as the one conducted by Garg et al. [3] in which 100 patients were analyzed of which 99% had type 2 DM and only 1% had type 1 DM.

Macro- and microangiopathy contribute significantly to the occurrence of com-plivations in diabetes. In patients with diabetes, there is an increase in vascular per-meability, a decrease in the sympathetic vascular response and a decreased ability to res-pond to heat and hypoxic stress [26,29,30,31,32]. In association with large vessel atherosclerosis, these microvascular abnormalities contribute to the formation of diabetic ulcers. In addition, in diabetes, loss of sensory innervation of the skin occurs, predisposing patients to infection and lesions [27]. Loss of neuroinflammatory signaling cells plays a determining role in the lack of healing of lower extremity ulcers [28,33,34,35,36].

The lesions strongly associated with diabetes mellitus were analyzed, with a predominance of diabetic foot (19 patients), followed by necrobiosis lipoidica—7 patients, with bullosis diabeticorum and acanthosis nigricans occurring in 6 and 5 patients respectively out of 103. A study conducted by Trihan et al. [33], which included 213 patients with DM shows a similar number of patients with necrobiosis lipoidica—5 patients, 4 with acanthosis nigricans and 3 with bullosis diabeticorum.

Impairments of immunoregulatory mechanisms may also occur in diabetes. Hyper-glycaemia and ketoacidosis impair chemotaxis, phagocytosis and bactericidal activity of white blood cells. This has changed dramatically with improved blood glucose control and antibiotic use. Despite these improvements, certain infections, such as otitis externa, necrotic infections and mucormycosis, occur more frequently in patients with diabetes [2,28,33,34]. A study conducted by Niaz et al. [34] found a predominance of bacterial infec-tions (26%), followed by fungal infections (22%), and we can correlate it with our study where bacterial infections also predominated (in 33% of patients) followed by fungal infections (31%).

Other DM-associated dermatoses were present in the group of patients presented in varying proportions, namely psoriasis (11 patients), lichen planus (9 patients) or granul-oma annulare (4 patients). The study conducted by Khokaro et al. [35] shows an association of the same conditions, with lichen planus and granuloma annulare being present in 4 patients each. Similar associations were also obtained by Mahajan et al. [36]—3 patients with psoriasis, 3 with bullous dermatoses, 2 patients with lichen planus.

No patients included in the current study experienced skin reactions to antidiabetic therapy-insulin or oral antidiabetics.

Limitations of this study are represented by the omission of certain DM-associated conditions as it is a retrospective study. There are also underdiagnosed manifestations that go unnoticed by the dermatologist’s eye.

## 5. Conclusions

Based on the present study and on those in the literature, we can state that skin is one of the main targets of diabetes complications. The numerous manifestations at this level show the complexity of the pathogenic mechanisms of DM and point to the importance of providing optimal care for the diabetic patient in order to increase their quality of life and prevent severe complications.

In view of the early manifestation of these complications, it is very important for the dermatologist to identify them in order to raise the suspicion of DM in patients not yet diagnosed.

## Figures and Tables

**Table 1:** Skin conditions associated with diabetes mellitus.

Manifestations Strongly Associated with DM *	Non-Specific Signs and Symptoms Associated with DM *	Dermatological Disorders Associated with DM *
acanthosis nigricans diabetic dermopathy diabetic foot limited joint mobility bullosis diabeticorum scleredema diabeticorum necrobiosis lipoidica	palmar erythema rubeosis faciei diabeticorum ichthyosis-like appearance of the tegument of the calves cutaneous xerosis eruptive xanthomas acrochordons diabetes-associated pruritus keratosis pilaris pigmented purpuric dermatoses yellow nail and skin syndrome onychocryptosis	generalized granuloma annulare psoriasis lichen planus vitiligo hidradenitis suppurativa

* DM = diabetes mellitus.

**Table 2:** Age and sex distribution of patients.

No.	Age (Years)	No. of Patients (n = 103)			Percentage (%)
		Female	Male	Total	
		60	43	103	
1	30–39	1	1	2	1.9
2	40–49	3	4	7	6.8
3	50–59	14	11	25	24.3
4	60–69	21	16	37	35.9
5	≥70	21	11	32	31.1

**Table 3:** Distribution by diabetes type.

		No. of Patients (n = 103)		
	Female	Male	Total	Percentage (%)
T1D	1	1	2	1.9
T2D	59	42	101	98.1

**Table 4:** Distribution by manifestations strongly associated with diabetes.

Lesion Type	No. of Patients		Chi2 Test		Percentage (%)
	Female	Male	*p*	Total	
Diabetic foot	7	12	0.037	19	18.4
Necrobiosis lipoidica	5	2	0.466	7	6.8
Bullosis diabeticorum	2	4	0.204	6	5.8
Acanthosis nigricans	1	4	0.077	5	4.9

**Table 5:** Non-specific cutaneous signs and symptoms associated with DM.

Event	No. of Patients		Chi2 Test		Percentage (%)
	Female	Male	*p*	Total	
Xerosis	28	16	0.341	44	42.7
Pruritus	7	9	0.203	16	15.5
Achrocordons	7	8	0.327	15	14.5
Rubeosis faciei	3	3	0.674	6	5.8

**Table 6:** Types of skin infections in patients with DM.

Type of Infection		No. of Patients	Chi2 Test		Percentage (%)
	Female	Male	*p*	Total	
A. Bacterial	12	23	0.001	35	33.9
Cellulitis	7	12	0.037	19	18.4
Folliculitis	4	9	0.032	13	12.6
Erythrasma	1	2	0.377	3	2.9
B. Fungal	46	35	0.565	81	78.6
Tinea pedis	15	17	0.118	32	31.1
Tinea corporis + cruris	20	8	0.099	28	27.1
Oral candidiasis	9	5	0.624	14	13.6
Genital candidiasis	2	5	0.101	7	6.8

**Table 7:** Other dermatological diseases associated with DM.

Disease	No. of Patients (n = 103)		Chi2 Test		Percentage (%)
	Female	Male	*p*	Total	
Other diseases *	12	7	0.633	19	18.4
Psoriasis	7	4	0.703	11	10.6
Lichen planus	6	3	0.514	9	8.7
Granuloma annulare	3	1	0.491	4	3.9

* Bullous dermatoses, lupus, morphea, and dermatomyositis.

## Data Availability

Not applicable.
